# Environmental Records from Great Barrier Reef Corals: Inshore versus Offshore Drivers

**DOI:** 10.1371/journal.pone.0077091

**Published:** 2013-10-18

**Authors:** Benjamin D. Walther, Michael J. Kingsford, Malcolm T. McCulloch

**Affiliations:** 1 Marine Science Institute, The University of Texas at Austin, Port Aransas, Texas, United States of America; 2 Research School of Earth Sciences, Australian National University, Canberra, Australian Capital Territory, Australia; 3 Australian Research Council Centre of Excellence for Coral Reef Studies, School of Marine and Tropical Biology, James Cook University, Townsville, Queensland, Australia; 4 Australian Research Council Centre of Excellence for Coral Reef Studies, School of Earth and Environment, The University of Western Australia, Crawley, Western Australia, Australia; University of New South Wales, Australia

## Abstract

The biogenic structures of stationary organisms can be effective recorders of environmental fluctuations. These proxy records of environmental change are preserved as geochemical signals in the carbonate skeletons of scleractinian corals and are useful for reconstructions of temporal and spatial fluctuations in the physical and chemical environments of coral reef ecosystems, including The Great Barrier Reef (GBR). We compared multi-year monitoring of water temperature and dissolved elements with analyses of chemical proxies recorded in *Porites* coral skeletons to identify the divergent mechanisms driving environmental variation at inshore versus offshore reefs. At inshore reefs, water Ba/Ca increased with the onset of monsoonal rains each year, indicating a dominant control of flooding on inshore ambient chemistry. Inshore multi-decadal records of coral Ba/Ca were also highly periodic in response to flood-driven pulses of terrigenous material. In contrast, an offshore reef at the edge of the continental shelf was subject to annual upwelling of waters that were presumed to be richer in Ba during summer months. Regular pulses of deep cold water were delivered to the reef as indicated by *in situ* temperature loggers and coral Ba/Ca. Our results indicate that although much of the GBR is subject to periodic environmental fluctuations, the mechanisms driving variation depend on proximity to the coast. Inshore reefs are primarily influenced by variable freshwater delivery and terrigenous erosion of catchments, while offshore reefs are dominated by seasonal and inter-annual variations in oceanographic conditions that influence the propensity for upwelling. The careful choice of sites can help distinguish between the various factors that promote Ba uptake in corals and therefore increase the utility of corals as monitors of spatial and temporal variation in environmental conditions.

## Introduction

Charles Darwin described the remarkable structural complexity and development of coral reefs while noting their dominance in clear, nutrient-poor waters [1]. Subsequent decades have brought considerable attention to explaining “Darwin's Paradox” of how biologically diverse coral reef systems can persist in oligotrophic environments. Substantial progress has been made in unravelling the importance of physical oceanography [2], structural complexity, symbioses and contributions of dominant taxa to nutrient budgets of reef communities. Although the potential importance of nutrient inputs from river outflow and upwelling were identified in early studies [3], subsequent paradigms of reef nutrient budgets assumed these systems were self-sustaining [4]. However, more recent workers have reaffirmed that periodic inputs of allochthonous material is a critical mechanism for not only sustaining reefs [5], [6] but in many coastal systems a major cause for degradation and loss of coral cover and diversity [7], [8]. Thus while major advances have been made in describing circulation and transport of water masses and associated materials in reef systems [9], [10], [11], validations of the periodicity, intensity and magnitude of delivery of nutrient-rich events at the individual reef scale are needed to fully understand the role of both temporal fluctuations and threshold-limiting responses to nutrient delivery events.

Coral reefs are dynamic systems that are subject to both high and low frequency fluctuations in environmental parameters such as salinity, temperature, terrigenous sedimentation and nutrient concentrations. These fluctuations can be seasonal, as for temperature, as well as pulsed on short temporal scales, as for storm-driven precipitation and coastal flooding. Further, the magnitude of these fluctuations often varies on inter-annual and even inter-decadal scales in response to climatic cycles and stochasticity. Often, the mechanisms driving temporal variation in coral reefs are not synoptic across the continental shelf, and heterogeneity in the dominant environmental forcing can depend on distance from shore. Importantly, the impacts of these fluctuations vary depending on their frequency and magnitude. For instance, flood-related delivery of terrigenous nutrients can help drive new production in oligotrophic waters [12], however excess nutrients and accompanying sedimentation can increase turbidity and lower water quality, potentially leading to habitat degradation and loss of coral diversity [7]. Conversely, reefs in locations not influenced by terrigenous runoff depend on nutrient inputs from other sources. In these locations upwelling of deep, nutrient-rich waters are a potential mechanism to support such reefs. Indeed, primary productivity at reefs receiving dissolved and particulate nutrient influxes associated with upwelling can be more than double that in downwelling-dominated regions [13]. This dynamic led Wyatt et al. [5] to introduce the concept of an “ocean catchment” whereby upwelling-influenced reefs are dependent on oceanic water masses that supply nutrients and sustain local production. Yet quantifying temporal and spatial fluctuations in either terrigenous or oceanic influx at individual reefs remains a challenge.

A salient feature of the Central Great Barrier Reef (GBR) is the lateral stratification of along-shelf currents. Nearshore coastal reefs are impacted by flooding and land runoff, which delivers terrigenous material and low salinity water in riverine plumes that extend from the river mouth [14]. Although floods follow a monsoonal seasonal cycle in this region, inter-annual variation in precipitation means that the magnitude of flooding can vary significantly on annual time scales. Further, riverine flood plumes are typically restricted to inshore regions and are usually steered northward close to shore [12]. This dynamic is due to the bifurcation of the South Equatorial Current (SEC) at approximately 14–19°S into the northward flowing Coral Sea Coastal Current (CSCC) and the southward flowing East Australian Current (EAC). For much of the year, the dominant offshore currents are northward or southward flowing, with limited cross-shelf transport [15]. Inshore flood plumes are constrained along the coast and steered northward due to their buoyancy, Coriolis-driven forcing and wind stress [12]. As a result, the influence of terrigenous material rarely propagates beyond the 40 m isobath to offshore reefs on the edge of the continental shelf [16], [17], [18].

In contrast, reefs at the edge of the continental shelf do not see the effects of flood plumes and are instead subject to seasonal upwelling of cold water onto the shelf due to a variety of processes including internal tides and wind-driven shelf waves. Variation in upwelling intensity may be controlled by large-scale oceanographic and climatic processes such as ENSO and fluctuations in the location of the SEC bifurcation, both of which alter the relative thermocline depth and therefore the ease with which deep water can be brought onto the shelf. Because offshore reefs at the Central GBR are far removed from the influence of flood plumes, it has been suggested that upwelling at the continental shelf edge is a significant source of nutrients to these outer reef habitats [19], [20].

Here we present results from a seawater sampling program combined with geochemical records preserved in coral skeletons to reconstruct and help identify the spatially divergent mechanisms driving environmental variability at nearshore and offshore reefs. In addition to multi-year assessments of water column properties using *in situ* monitors and a program of temporal and spatial water sampling, we relied on the chemical properties of incrementally accreting skeletons of massive *Porites* corals to identify temporal fluctuations in environmental conditions. This approach rests on previous observations that ambient dissolved water concentrations of barium (Ba) are strongly influenced by either terrigenous runoff or upwelling of nutrient-rich water, as Ba concentrations are relatively enriched in both fresh and deep marine waters [21], [26], [22]. Because Ba is effectively incorporated into growing coral skeletons, retrospective analyses of coral Ba across skeletal increments allows us to reconstruct environmental forcing over time. Our comparison of inshore and offshore reefs in the Central GBR capitalizes on the very different hydrographic dynamics across the continental shelf, providing strong contrasts in forces that drive across-shelf dynamics and nutrient delivery.

## Materials and Methods

### Ethics statement

Entry into sampling zones and collection of water and coral specimens were done under approved procedures from the Australian Government Great Barrier Reef Marine Park Authority (GBRMPA Permits G07/25134.1, G07/24374.1, and G08/26596.1). This study did not involve endangered or protected species.

### Study sites

**Figure 1:**
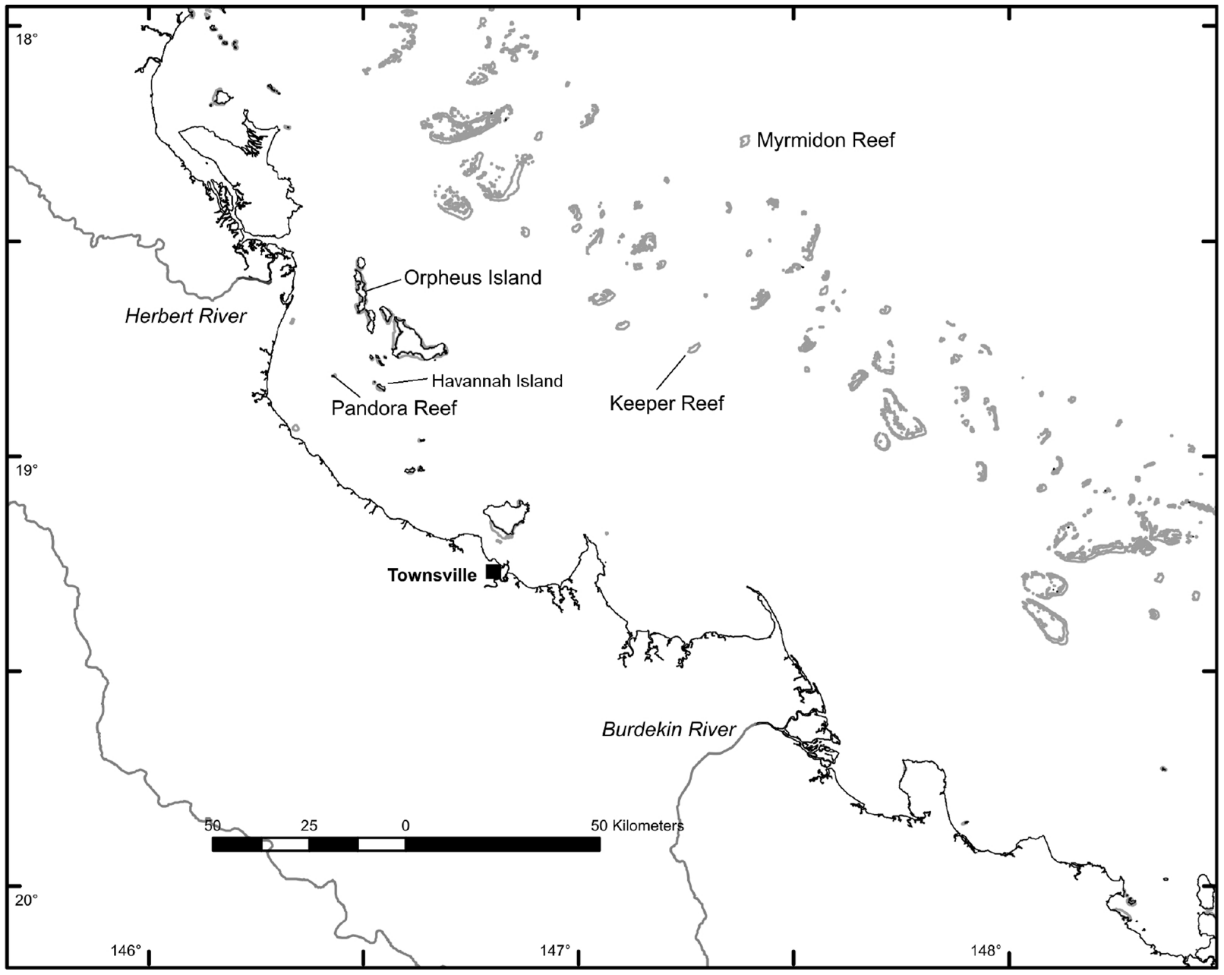
Location map. Map showing location of Havannah/Pandora/Orpheus island complex, Myrmidon reef, and Herbert and Burdekin rivers in the central section of the Great Barrier Reef, Australia.

The inshore coral core was retrieved from the fringing reef adjacent to Havannah Island on the Central GBR (Figure 1). Reefs at the Havannah/Palm/Orpheus Island complex sit in the path of low salinity and high terrigenous content plumes that are advected northward from the Burdekin River mouth during periods of flooding. These plumes are constrained to the inner continental shelf by strong alongshore currents that limit mixing and transport of terrigenous material across the shelf. The inshore Havannah core was drilled from a site on the eastern side of the island at a depth of 2–3 m (18°50.378; 146°33.010) The offshore core was retrieved from Myrmidon Reef, which sits approximately 100 km off the coast of Queensland on the edge of the continental shelf (Figure 1). This coral was at a site located on the forereef slope at a depth of 25 m (18°15.682, 147°22.592). Temperature loggers were deployed for multiple years at reefs at Orpheus Island, Havannah Island, and Myrmidon Reef. Loggers recorded values at a 1 hour frequency and were retrieved annually. Although complete temporal coverage of the study period was not achieved in all locations due to occasional logger loss or malfunction, the overall temperature structure of both locations was nevertheless well constrained.

### Water sampling & analysis

Duplicate water samples were taken from three surface sites at monthly intervals between 500 m and 2 km west of Orpheus Island. Duplicate water samples were also taken at 50–100 m intervals up to a maximum depth of 150 or 200 m at locations 2 km east of Myrmidon Reef. At both locations, samples were taken using polypropylene syringes and filtered through 0.45 µm MF-Millipore™ filters and acidified to 1% with 20% ultrapure HNO_3_ prior to analysis at the Advanced Analytical Centre (James Cook University). Samples were diluted 10 fold prior to Ba measurements on a Varian 820-MS inductively coupled plasma mass spectrometer (ICP-MS) using H_2_ as a CRI gas at a flow rate of 120 ml/min. Concentrations of dissolved Ca were determined by a Varian Liberty Series II ICP-AES after analyses on the 820-MS. Multi-element standard solutions (1 and 5 ppb) were used to calibrate the instruments while 20 ppb of Y and In were added as internal standards to control for instrumental drift and matrix effects. A CASS-4 sea water certified reference material (CRM) was diluted 10 fold, spiked with 1 ppb of the multi-element standard, and analysed every 20 samples for quality control and used to subtract backgrounds from all analysed samples. Long term analytical precision (relative standard deviation) was estimated to be 3.6% for Ba/Ca.

### Coral sampling and analysis

The Havannah core was 64 cm long, while the Myrmidon core was 21 cm long. Sample preparation followed Sinclair et al. [23] with modifications. For the inshore Havannah site other coral records prior to 2005 were also available from previous studies [26], [27] and are shown in the supplementary section (Fig. S3). Cores were cut into 7 mm thick slices, after which they were x-rayed to visualize annual density variations [24]. Slices were cut into 95×25 mm pieces to fit the laser sampling cell, making sure to cut pieces only part-way through with the sectioning blade, after which pieces were pulled apart manually [25]. This ensured that the ablation surface had minimal/zero loss of skeletal material due to cutting. Where variable growth trajectories necessitated switching to a new growth axis, samples were overlapped to contain equivalent growth increments and enable subsequent stitching of contiguous elemental data from pieces to result in a continuous time series across all increments. All pieces were cleaned in a bath of ultra pure Milli Q water with an ultra-sonic probe and dried in an oven at 48°C overnight.

Laser ablation inductively coupled plasma mass spectrometry (LA-ICP-MS) was used to quantify elemental intensities with a 193 nm ArF excimer laser coupled to a Varian 820 ICP-MS at the Research School of Earth Sciences laboratory (Australian National University). Ablated material was delivered to the ICP-MS via a He carrier gas. Prior to analysis, laser tracks were pre-ablated to remove surface contaminants. Ablation was carried out with a rectangular 40×400 µm slit with the narrow width parallel to the growth axis, a 40 µm per second span speed, 5 Hz repetition rate, 50 MJ of energy, and a 50% partially reflecting mirror. Analyses of each piece were bracketed at the beginning and end by measurements of glass NIST 614 certified reference material and an in-house pressed powder coral standard [26]. Sixty seconds of background counts were collected before and after each block of the standards and coral piece for offline subtraction of background intensities. During ablation, the isotopes ^43^Ca, ^86^Sr and ^138^Ba were monitored continuously. Elemental intensities were normalized to Ca and corrected for elemental bias using interpolations of the pressed coral standard following Fallon et al. [27] to obtain molar ratios. External precisions based on repeated measurements of NIST 614 over all analysis days (*n* = 60) were 3.3% for Sr/Ca and 4.3% for Ba/Ca. Data were smoothed with a 10-point averaging box filter to remove high frequency variation deriving from instrumental instability and counting statistics [23], [28].

Once coral pieces were analysed, elemental time series were stitched together by joining consecutive series and removing redundant increments for pieces with overlapping sections. A chronology was then added to the time series by using complementary methods to establish dating checkpoints. First, inshore corals were examined under UV light, as the intensity of luminescent banding in skeletons correlates with flooding intensity [29]. Also, previous validations have established temperature relationships for some coral elemental ratios, such that Sr/Ca is inversely proportional to ambient temperature [23], [30]. Low winter temperatures thus correspond to peaks in Sr/Ca. We used the geochronological software AnalySeries [31] to compare coral Sr/Ca peaks and troughs to monthly SST measurements from 1°×1° grids centred on the reef of interest from the NOAA Reyn_SmithOIv2 data set [32]. Time was then interpreted linearly between summer minima and winter maxima at a monthly resolution. Assigned dates were crosschecked against the UV luminosity banding deriving from flood events for the Havannah core and x-ray images for both cores for verification.

### Partition coefficients

Comparisons were made between monthly dissolved Ba/Ca ratios sampled at Orpheus Island between 2006-early 2008 and the Havannah coral Ba/Ca ratios for the same time period. The distribution, or partition, coefficient was calculated as D_Ba_ = (Ba/Ca)_coral_/(Ba/Ca)_seawater_ following Lea et al. [33].

### Wavelet spectral analysis

Time series of coral Ba/Ca were investigated with a wavelet analysis to identify temporal variations of spectral power [34]. This procedure is analogous to a windowed Fourier transform and explicitly compares wavelets of different periods across a given time series to identify localized variations in spectral power. Further, the method of Torrence & Compo [34] allows statistical significance testing of power spectra at specified probabilities above designated background noise characteristics.

Both Havannah and Myrmidon Ba/Ca time series were analysed with a Morlet wavelet with an ω_0_ (number of oscillations in the wavelet) of 6 and a scale width s_0_ (smallest resolvable scale) of 0.25 years. Spectral contours were defined at 75%, 50%, 25% and 5% wavelet power levels, which were defined separately for each coral time series. Significance contours were calculated at p = 0.05 significance level above a red-noise (autoregressive lag-1) background spectrum. Plots of spectral power were overlain with a cross-hatched “cone of influence” area indicating reduced ability to detect power due to errors associated with edge effects, which dominate at the beginning and end of the time series as well as at lower frequencies.

## Results

### Temperature time series

**Figure 2:**
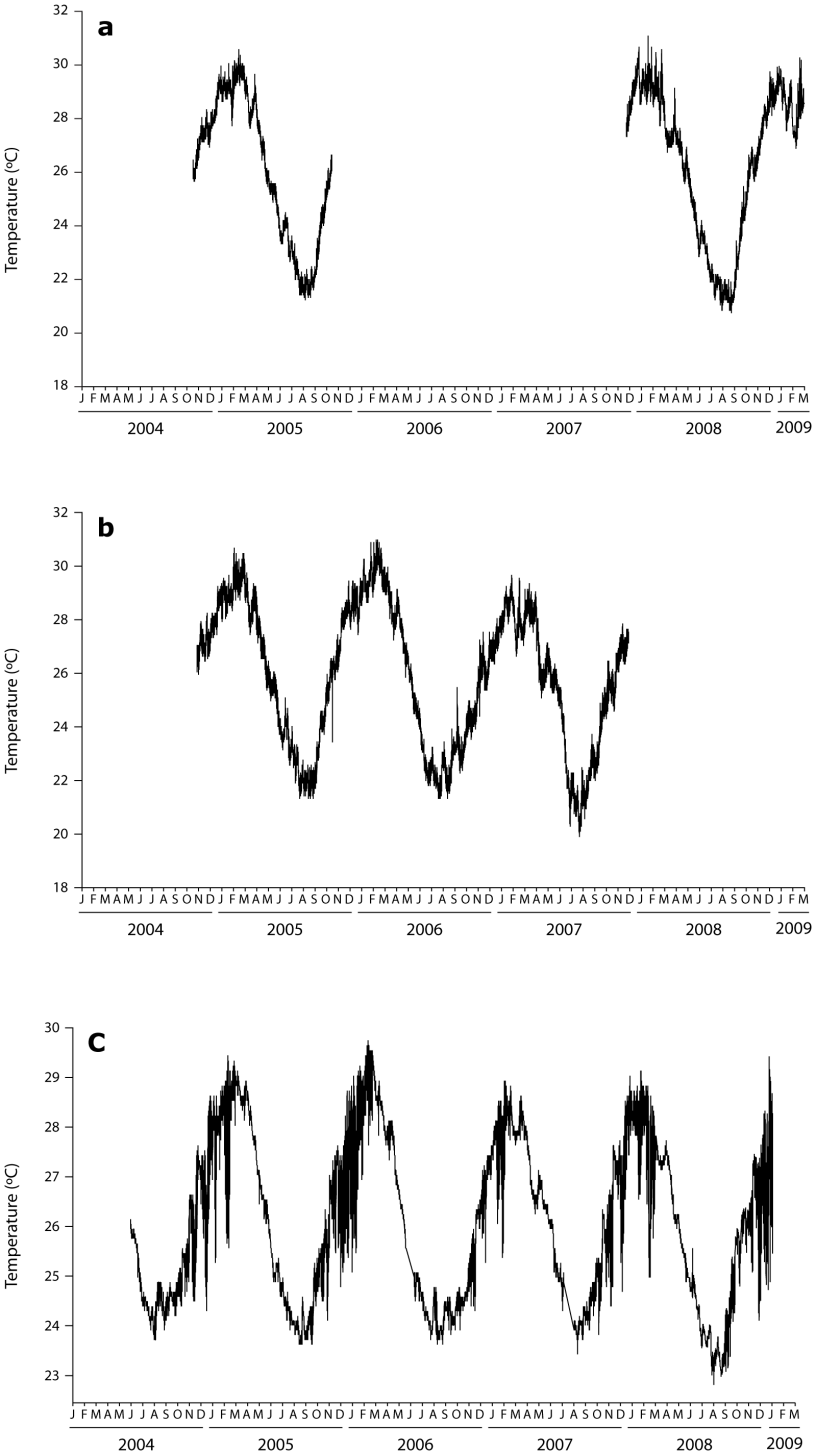
Water temperatures. Temperature data from *in situ* loggers for the period of 2004–2008 (some years with missing data) at a) Havannah Island, b) Orpheus Island and c) Myrmidon Reef.

Temperatures in all regions showed strong seasonal oscillations (Figure 2), with maxima in the austral summer (January–February) and minima in winter (July–August). At inshore reefs, summer maxima were near 30°C and minima near 21°C. Seasonal ranges were diminished at the offshore site, with maxima of near 29°C and minima near 22–23°C. In marked contrast to the inshore temperature time series, the forereef section of Myrmidon experienced significant temperature excursions during spring and summer when cold water intruded onto the shelf break. Temperature depressions were as high as 3°C, and while individual events were discrete and generally related to the state of the tide, with sustained excurisions of lower than average temperatures that lasted hours to days, the periods of upwelling events lasted weeks to months.

### Water chemistry

**Figure 3:**
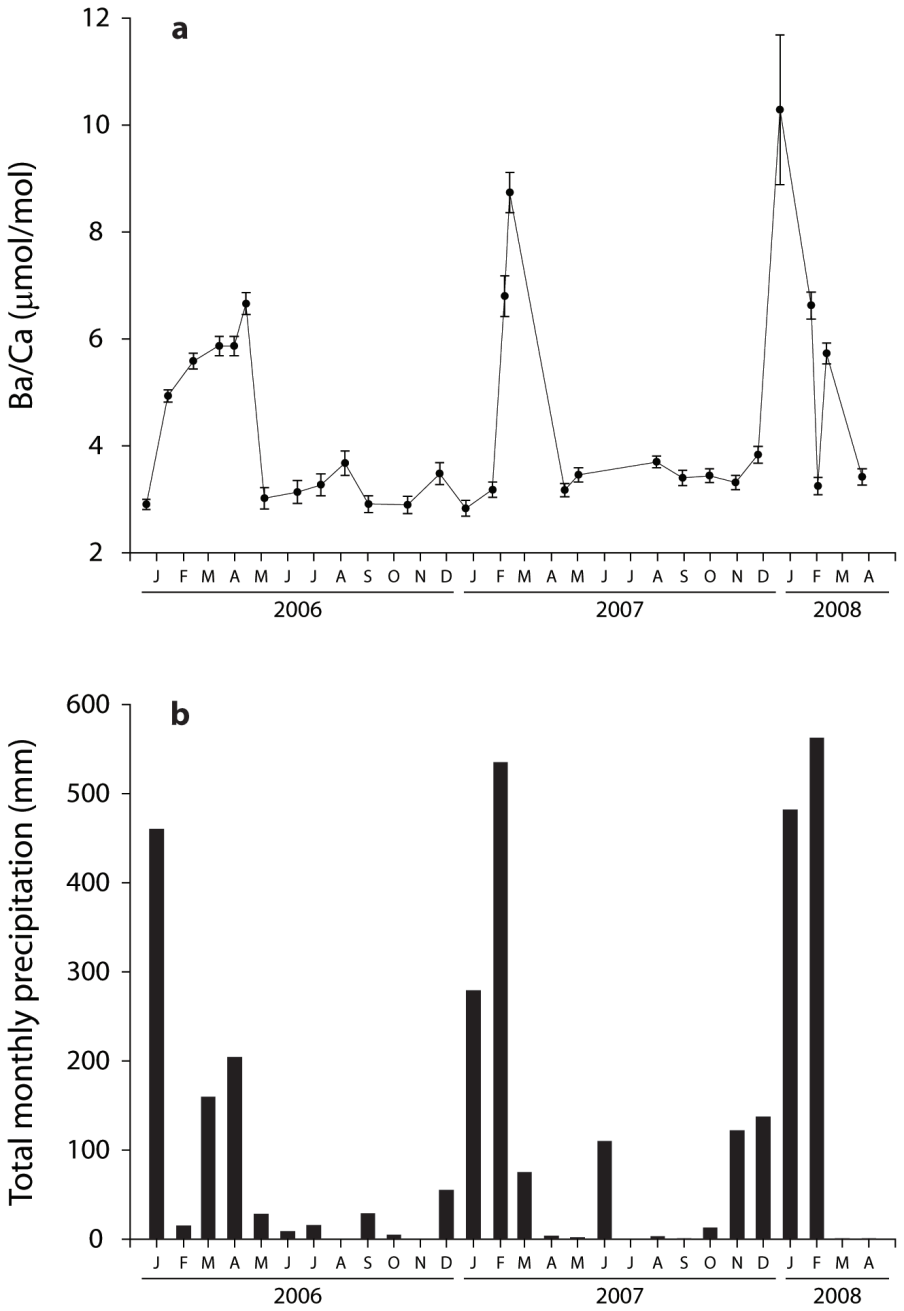
Inshore water chemistry and precipitation. Time series of flood signals for inshore reefs reflected in a) ambient dissolved Ba/Ca concentrations (mean ±1 SD) sampled regularly at Orpheus Island from 2006 through mid-2008 and b) total monthly precipitation measured at the Townsville Airport (Bureau of Meteorology station 32040).

Ambient dissolved water Ba/Ca ratios at the inshore site varied in accordance with seasonal flooding (Figure 3). Water Ba/Ca peaked in Jan–March reflecting monthly precipitation patterns and therefore the onset of riverine outflow to inshore reefs. The mean ambient Ba/Ca ratio for the entire sampled period was 4.3±1.9 µmol/mol, while background Ba/Ca values (May–November) were between 2.8–3.8 µmol/mol. Peak Ba/Ca ratios of 10.3±1.4 µmol/mol were observed in December 2007.

**Figure 4:**
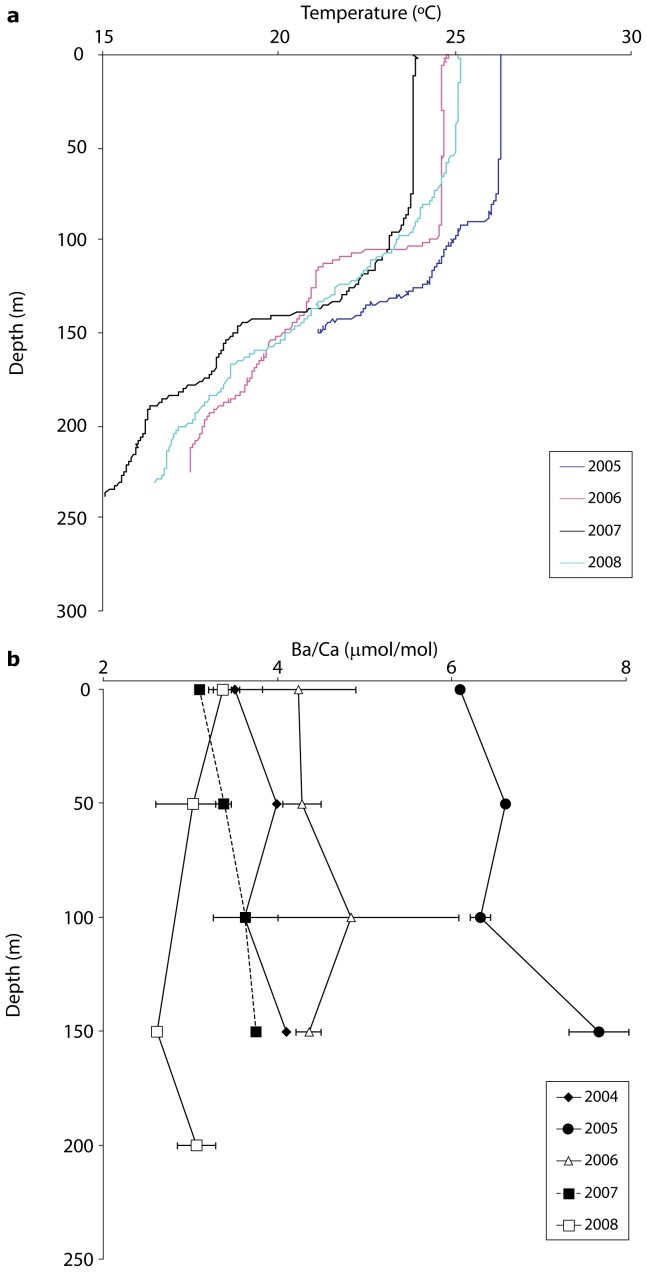
Offshore water chemistry. Depth transects at Myrmidon Reef of a) temperature and b) ambient dissolved Ba/Ca concentrations (mean ±1 SD). Error bars are smaller than symbols in some cases.

At the offshore site, depth profiles of temperature and Ba/Ca values taken during the austral winter over several years showed predicted patterns of water column stratification and low surface Ba/Ca values as expected in winter (Figure 4). Surface temperatures ranged from 24–26°C with a thermocline at 75–100 m depth. With the exception of 2008, surface Ba/Ca values were lowest, as would be expected from the typical nutrient-like distribution of Ba in oceanic waters due to scavenging and export from surface waters [21]. Surface Ba/Ca values ranged from 3.11 µmol/mol in 2007 to 6.09 µmol/mol in 2005, and Ba/Ca values never exceeded 8.0 µmol/mol in the sampled portion of the water column (≥200 m). There was little evidence for elevated Ba/Ca values within the upper 200 m in winter. Higher levels of Ba/Ca, therefore, would be expected at depths greater than 200 m at this time.

### Coral chemistry

**Figure 5:**
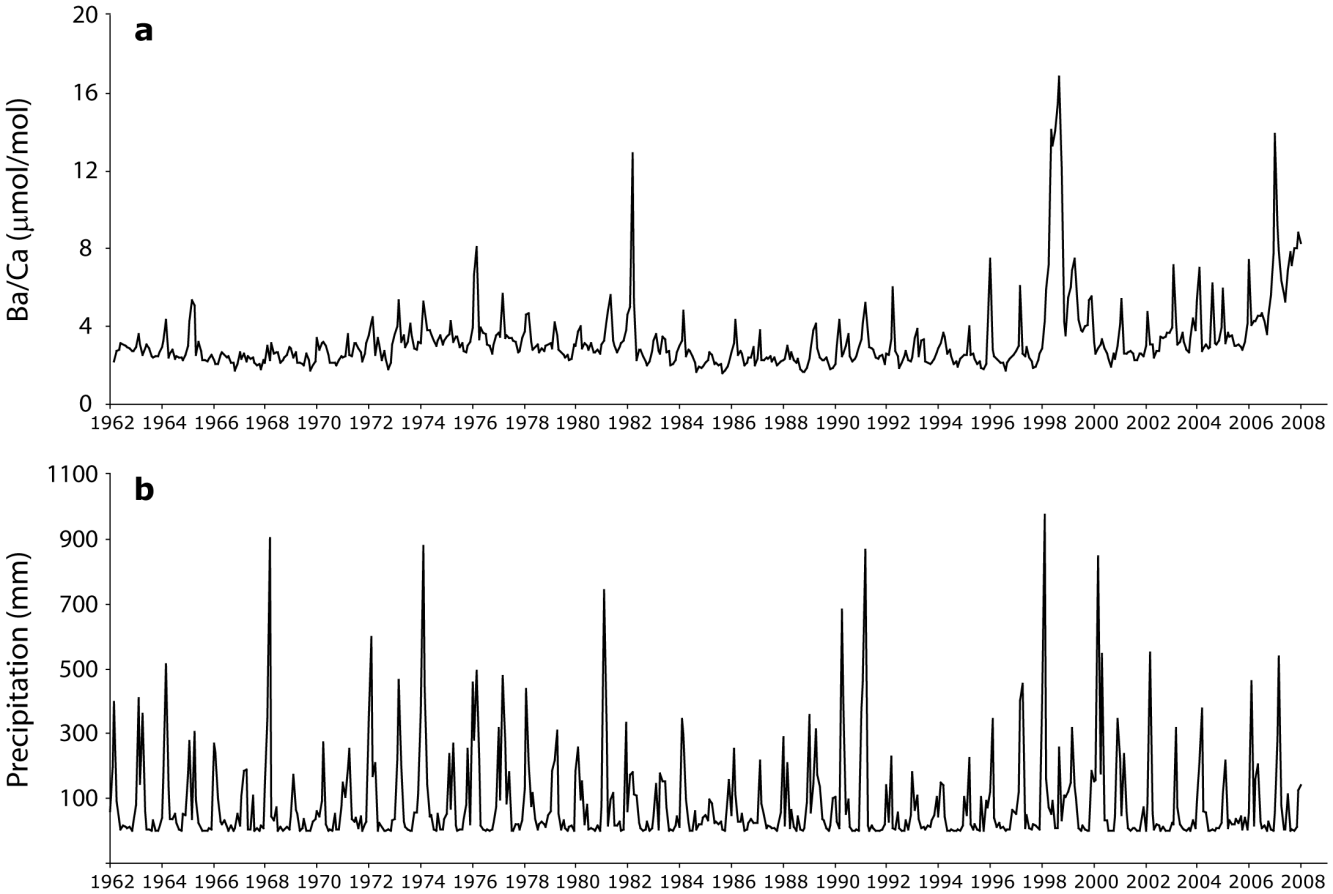
Inshore coral chemistry and precipitation. Time series of a) Havannah coral Ba/Ca ratios for the period 1962–2008 and b) historical monthly total precipitation measured at the Townsville Airport (Bureau of Meteorology station 32040).

**Figure 6:**
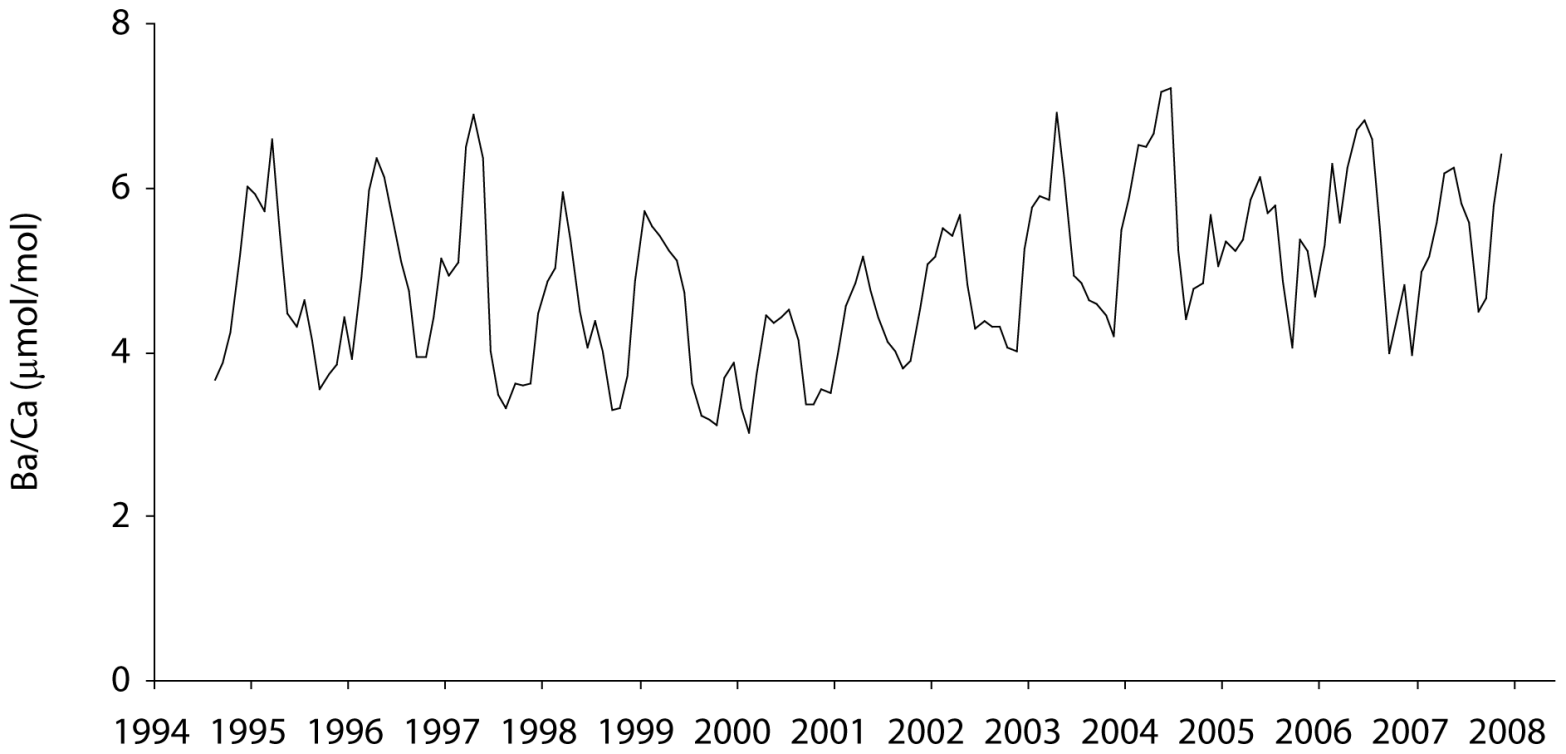
Offshore coral chemistry. Time series of Myrmidon coral Ba/Ca ratios for the period 1994–2008.

As expected, Sr/Ca values varied inversely with SST in the inshore Havannah coral (Figure S1). In contrast, the Ba/Ca profile from the Havannah coral showed oscillations that corresponded to occurrence of flooding events which occurs at approximate annual timescales but with highly varying intensity(Figure 5). Fluctuations in coral Ba/Ca were observed for the entire 1962–2008 time series, with peaks typically occurring between January and March. The highest peaks were observed in 1997–98 (16.9 µmol/mol) and 1981–82 (12.9 µmol/mol) that corresponded to intense El Niño phases and major flooding. For the period of 2006 through early 2008, the mean coral Ba/Ca ratio was 6.4±2.4 µmol/mol (1 SD), resulting in an average partition coefficient D_Ba_ of 1.7±0.8. The Ba/Ca profile from the offshore Myrmidon core showed strong periodic annual oscillations consistent with summertime upwelling and delivery of Ba-rich deep waters to this shelf-edge region (Figure 6). Although Sr/Ca values were inversely related to SST in the Myrmidon core, Ba/Ca values were positively related to SST (Figure S2). This suggests that the oscillatory Ba/Ca values were not due to seasonal or upwelling temperature fluctuations, as both Ba/Ca and Sr/Ca would show similar negative relationships with SST in that case as both Sr and Ba have temperature dependent distribution coefficients that are >1 (see discussion). The amplitude and duration of Ba/Ca peaks also varied inter-annually, perhaps indicative of inter-annual differences in upwelling intensities.

**Figure 7:**
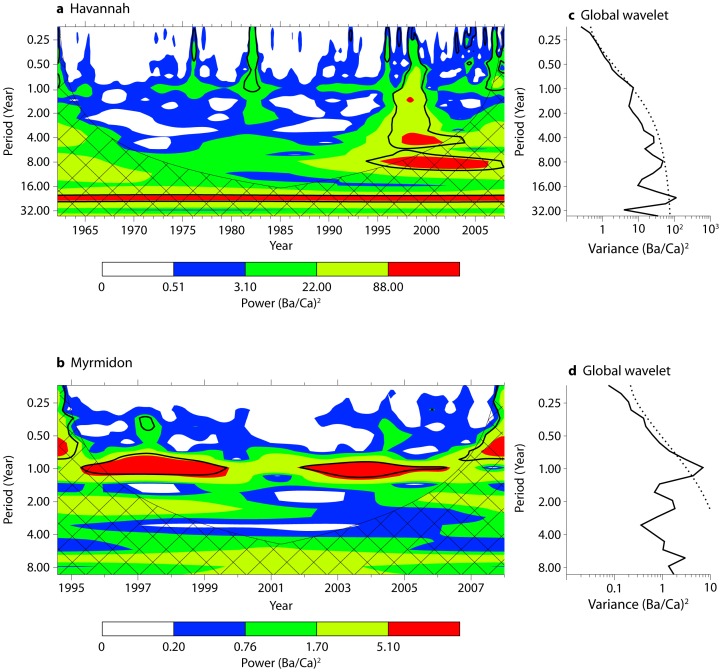
Coral chemistry wavelet power spectra. Temporally-explicit wavelet power spectra for a) Havannah and b) Myrmidon coral Ba/Ca. Contour levels are calculated separately for each time series and represent the 75%, 50%, 25% and 5% power contours, respectively. The solid black contour is the p = 0.05 significance level above a red-noise background spectrum. The cross-hatched region is the cone of influence where edge effects may bias power calculations. The time-averaged global wavelet of the entire series for c) Havannah and d) Myrmidon are also shown. This output is analogous to a Fourier power spectrum [34]. The dashed line indicates the p = 0.5 significance level across different periods.

The wavelet power spectra reflected the region-specific dynamics of Ba pulses to reefs (Figure 7). At Havannah, power spectra were complex across multiple periods. A strong annual signal (i.e. power) was generally consistent across the time series, which at times exceeded statistical significance. However, power elevations were observed at other longer periods, reflecting the high degree of inter-annual variability in magnitudes of flood-related Ba/Ca spikes that concord with longer period oscillations. There was a noticeable elevation of power at the 4–10 year period, perhaps corresponding to ENSO oscillations [35]. The 1997–98 Ba/Ca pulse resulted in widespread power elevations across periods, indicating that this event was more than simply an annual excursion and was aligned with multi-frequency oscillations. In contrast, the Myrmidon Ba/Ca profile exhibited intense power at the annual scale, which nearly always exceeded significance levels. Because of the shortness of the time series, longer period oscillations were more subject to edge effect bias, although power elevations were observed at the 6–7 year period, suggestive of ENSO oscillations.

## Discussion

Our results demonstrate the considerable spatiotemporal variation in external mechanisms that affect nutrient delivery and water chemistry across the continental shelf of the GBR. A persistent challenge in employing geochemical proxies in reconstructing environmental effects is determining the relative importance of endogenous versus exogenous factors that influence elemental ratios in biogenic carbonates. In order to be confident that our coral time series reflected delivery of dissolved Ba by river plumes or upwelling, alternative explanations for the observed patterns must be eliminated. An important component of this study was the water sampling program that allowed us to characterize dissolved Ba dynamics. As emphasized by Sinclair & McCulloch [36], direct measurements of dissolved Ba are rare yet critical for characterizing dynamics of terrigenous delivery to inshore reefs. Our monthly water sampling at Orpheus Island showed that pulses in ambient seawater Ba was highly responsive to the onset and magnitude of precipitation and riverine outflow events that led to the annual and inter-annual variation in the inshore coral Ba/Ca values. In addition, the high resolution inshore water sampling program allowed us to calculate a partition coefficient of 1.7±0.8 for the Havannah coral, which is within one standard deviation of the coral D_Ba_ value of 1.4±0.1 calculated by Lea et al [33] and 1.2 by Alibert et al. [37]. Conversely, concentrations of surface water Ba was generally low at Myrmidon reef at the surface in winter, and even higher dissolved Ba would be expected at depths greater than 200 m [38]. These water samples combined with the continuous temperature records allowed us to confidently characterize the spatially unique environmental dynamics of inshore and offshore reefs.

The influence of temperature on coral geochemistry must also be considered. As pointed out by Lea et al [33] and Gaetani and Cohen [39], an alternative explanation for seasonal cycles in coral Ba/Ca ratios is temperature-driven differences in precipitation efficiency, defined as the mass fraction of aragonite precipitated from the calcifying fluid, and therefore elemental incorporation dynamics leading to annual cycles concordant with seasonal temperature oscillations. However, precipitation experiments by Gaetani and Cohen [39] definitively show that both Ba/Ca and Sr/Ca in inorganic aragonite have negative relationships with temperature, meaning that maximal Ba/Ca and Sr/Ca values would be observed at wintertime if a temperature signal were to be observed. In contrast, our coral shows elevated Ba/Ca ratios (and minimal Sr/Ca ratios) during summer temperature maxima when upwelling intensity is greatest. In *Porites* the coral Ba/Ca record is indicative of upwelled dissolved Ba and not temperature.

The Ba/Ca pattern observed at the inshore Havannah coral agreed with previous analyses of cores from this region [26], [37], [40]. Strong step-function peaks in Ba/Ca reflecting the arrival of monsoonal rains and terrigenous material delivered to inshore reefs by wide-ranging river plumes. The inter-annual variability in Ba/Ca intensity reflected inter-annual fluctuations in the duration and intensity of rainy seasons. Unusually high peaks were observed in 1997–98 and 1981–82 that corresponded to intense El Niño phases and major flood events following extended periods of drought. This pattern was expected, given that drought-breaking floods deliver increased sediment loads to coastal reefs [26]. However, intense coral Ba/Ca peaks were not observed in all expected drought-breaking years, such as 1974 and 1968. Previously published coral records [26] from other locations at Havannah that cover this period 1960–2000 do show more significant elevations in Ba/Ca for some of these years (Figure S3). The lower magnitude than expected for a few of these peaks in the present record is likely due to the specific small-scale geographic positioning of the coral sampled here, such that an island “shadowing” effect limited the delivery of terrigenous material in some years. Micro-topography and small-scale circulation patterns can thus be an important factor in coral proxies. However, the Havannah record presented here is coherent with other published records in regards to periodicity and inter-annual intensity variations that indicate the dominant control of flood plume events on these inshore reefs.

In contrast to the inshore coral, the offshore Myrmidon coral showed seasonal oscillations in Ba/Ca that are attributed to upwelling. Our study is the first to suggest upwelling signals in corals from the GBR, and this pattern agrees with results from other workers using coral Ba/Ca as a proxy for upwelling in the Galapagos [33], [41], New Caledonia [42], [43], Papua New Guinea [44], [45], Tahiti [46], the Arabian Sea [47], northern Japan [27], and Venezuela [48]. Intriguingly, the Myrmidon coral showed sustained periods of elevated skeletal Ba/Ca over the course of the summer, despite the fact that upwelling events as indicated by temperature excursions were often pulsed and brief. Temperature loggers showed individual upwelling events occurred on the order of days, although some years (e.g. summer 2005–2006) saw extended periods of multiple upwellings over a few months. However, although individual upwelling events lasted for hours to days, the annual total number of upwelling days can be as high as 130 days at Myrmidon and other offshore reefs [49]. Sustained periods of high frequency upwelling events could repeatedly inject Ba-rich water into the reef over the austral summer to produce extended peaks of Ba in coral skeletons from these sites. Thus, unlike the typically punctuated onset of monsoonal rains affecting inshore reefs, upwelling phenomena are repeated and more extended in duration. In addition, coral Ba concentrations and luminescence patterns typically exhibit ‘extended tails’ after punctuated perturbations [36], [50] showing the persistence of high Ba/Ca low salinity waters following very major events. Extended tails after each discreet Ba pulse could lead to temporal averaging on timescales of weeks coupled with repeated and protracted inputs of Ba-rich water at upwelling and explain the extended seasonal elevations in coral Ba/Ca observed here, as it would act as a continuous smoothing process overlain on extended high frequency inputs of upwelled Ba.

Upwelling dynamics at the central GBR are primarily controlled by the relative depth of the thermocline at the edge of the continental shelf. Climatological and physical forces that modulate thermocline depth and therefore upwelling events are numerous and include internal waves, seasonal warming and cooling, wind forcing, tides, eddies, EAC and CSCC intensity, and cycles of ENSO and PDO. On a short-term seasonal basis, upwelling is predicted during summer months when thermoclines shoal to above 50 m [49]. On a longer-term basis, upwelling propensity and intensity are expected to be reduced during extended La Niña conditions when thermoclines in the western Pacific are depressed [51]. Teasing apart the relative effects of these combined forces is difficult, particularly when available time series are short relative to the period of oscillation for climatological cycles such as ENSO. In a rare multi-year analysis of upwelling on the outer Central GBR, Berkelmans et al. [49] demonstrated that upwelling frequency and intensity is strongly positively correlated to summer temperature on both a seasonal and inter-annual basis. The mechanistic link between the two was speculated to be the intensity of the poleward-flowing EAC, which would bring the thermocline closer to the surface and thereby allow easier onshore transport of sub-thermocline water. High EAC intensities typically occur in El Niño years. Coral chemical records can be of considerable use in identifying periodicity and intensity of climatological phenomena such as ENSO [52], [53]. In fact, our offshore coral Ba/Ca record showed indications of longer period oscillations that may reflect ENSO conditions, with apparent reductions in peak values for the period 1999–2002 during a La Niña phase [54], [55]. This pattern could not be conclusively established, however, given that our coral time series was only 13 years long, roughly corresponding to one ENSO oscillation. Longer coral Ba/Ca time series from outer shelf upwelling regions are necessary to validate this relationship, as has been observed with other coral proxies [56].

The potential importance of upwelling for supporting coral reefs and the GBR in particular was noted early by Orr [3], who observed nutrient enrichments at depth near the shelf break and observed, “A factor which may be of importance here is the upwelling of deep water rich in plant salts close to the Barrier” (p. 62). Several decades later, a series of papers in the 1980s expanded the quantification of upwelling contributions to nutrient influx and primary productivity in the Central GBR. In a rigorous survey of upwelling dynamics across the Central GBR, Andrews & Gentian [57] found that at Myrmidon reef during upwelling events nitrate concentrations were an order of magnitude higher and chlorophyll concentrations were approximately double compared to those found shoreward in the unstratified GBR lagoon. The relative magnitude of upwelled nutrients is not trivial for reef budgets. Recent comparisons of allochthonous inputs of nutrients to the Central GBR found that annually averaged flux inputs of upwelled P (27×10^6^ mol yr^−1^) is comparable to riverine inputs (27×10^6^ mol yr^−1^), while upwelled N (152×10^6^ mol yr^−1^) is nearly one quarter of riverine N input (660×10^6^ mol yr^−1^) to the Central GBR, with much of the remaining offshore N input being contributed by N-fixation [20]. Upwelled water can propagate around 50 kilometres inshore of the shelf break [57], indicating that these events deliver nutrients across the outer reef matrix and provide comprehensive delivery of organic sustenance to the outer GBR. However, further intrusion of upwelled materials across the entire shelf is minimal. In a box modelling approach to GBR nutrient budgeting, Furnas et al. [20] estimated that less than 5% of upwelled N or P intrudes across the shelf. Thus upwelling impacts are significant yet limited to the outer reef. Although large-scale upwelling events can be coherent at scales of several hundred kilometres [58], local variation in local submarine topography and current fields can lead to significant differences in upwelling frequency and intensity at individual reefs [15]. Furthermore, upwelling signals may be obscured in lagoonal habitats, where injected upwelled nutrients (and Ba) are rapidly transformed and allochthonous inputs may be obscured by local resuspension dynamics. As with the Havannah core, careful choice of locations for obtaining coral proxies is necessary for accurate interpretation. The use of coral chemical proxies to characterize local time series of upwelling dynamics offers a novel way to refine spatially explicit nutrient budgets, particularly when sampled corals are chosen carefully from forereef locations.

In summary, we used a combination of water sampling and coral chemistry analyses to demonstrate significant differences in the characteristics of pulsed allochthonous input events across the central GBR. Our water sampling program was unique and illustrated the dependence of ambient chemistry on either river flood plumes or upwelling depending on the distance from the coast. Importantly, we chose our sampling locations carefully such that we could distinguish between the effects of floods and upwelling on geochemical proxies in coral skeletons. An approach that combines long-term monitoring of water chemistry data and coral chemistry from carefully selected sites is necessary in order to fully assess the spatiotemporal variations of external inputs to coral reefs and progress towards closing the nutrient budgets of these dynamic systems.

## Supporting Information

Figure S1 **Havannah Sr/Ca and SST.** Regression between SST and Sr/Ca for Havannah coral core reported in this paper. The expected negative relationship is observed.
(TIF)

Figure S2 **Myrmidon Sr/Ca, Ba/Ca and SST.** Regressions between SST and a) Sr/Ca and b) Ba/Ca for the Myrmidon coral core reported in this paper. The regression with Sr/Ca is negative, as expected for seasonal oscillations in temperature. The regression with Ba/Ca is positive, which is opposite what would be expected if Ba/Ca values were controlled by temperature.
(TIF)

Figure S3 **Havannah Ba/Ca timeseries.** Portion of previously published [26] compilations of coral records retrieved from other locations at Havannah Island. Some major drought-breaking flooding events are more prominent in these records (e.g. 1968, 1974), likely due to island shadowing of plumes for the record presented in the main text.
(TIF)
