# Supplementary figures and images for: Environmental Records from Great Barrier Reef Corals: Inshore versus Offshore Drivers
Source: PLoS One. 2013 Oct 18;8(10):e77091. doi: 10.1371/journal.pone.0077091 (PMC3799737; doi:10.1371/journal.pone.0077091)

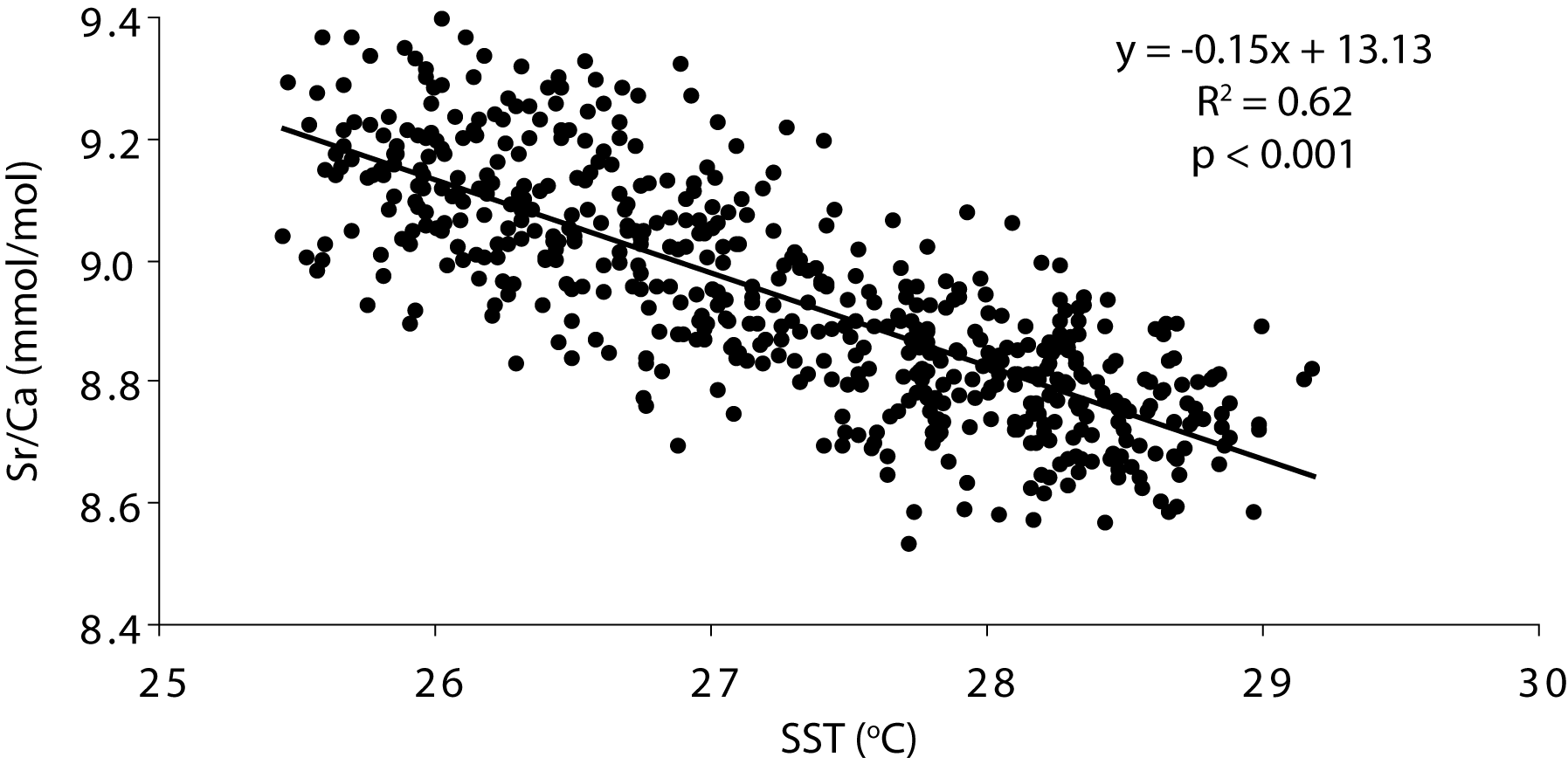

Supplement: Figure S1 — Havannah Sr/Ca and SST. Regression between SST and Sr/Ca for Havannah coral core reported in this paper. The expected negative relationship is observed. (TIF) [file pone.0077091.s001.tif]

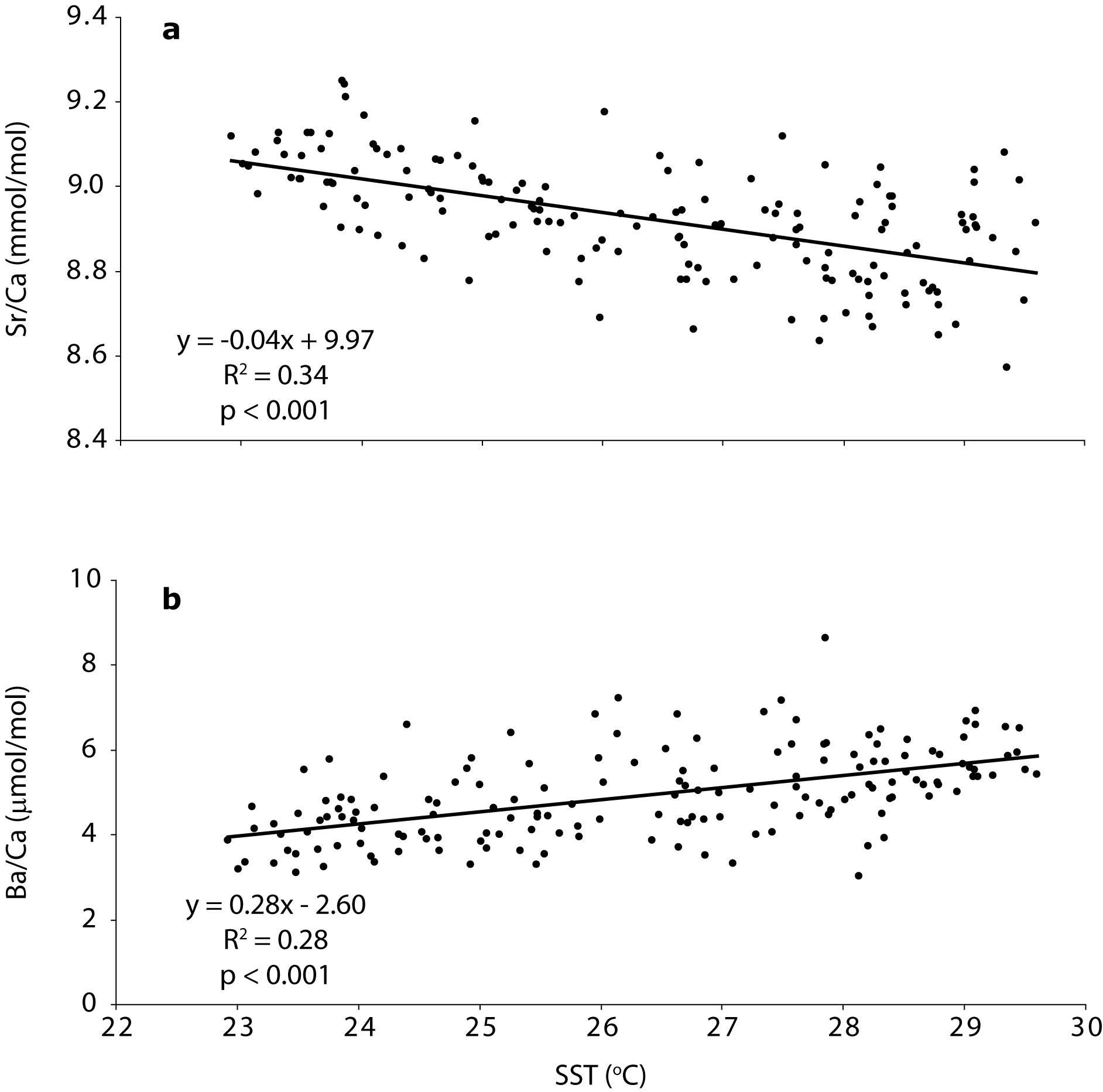

Supplement: Figure S2 — Myrmidon Sr/Ca, Ba/Ca and SST. Regressions between SST and a) Sr/Ca and b) Ba/Ca for the Myrmidon coral core reported in this paper. The regression with Sr/Ca is negative, as expected for seasonal oscillations in temperature. The regression with Ba/Ca is positive, which is opposite what would be expected if Ba/Ca values were controlled by temperature. (TIF) [file pone.0077091.s002.tif]

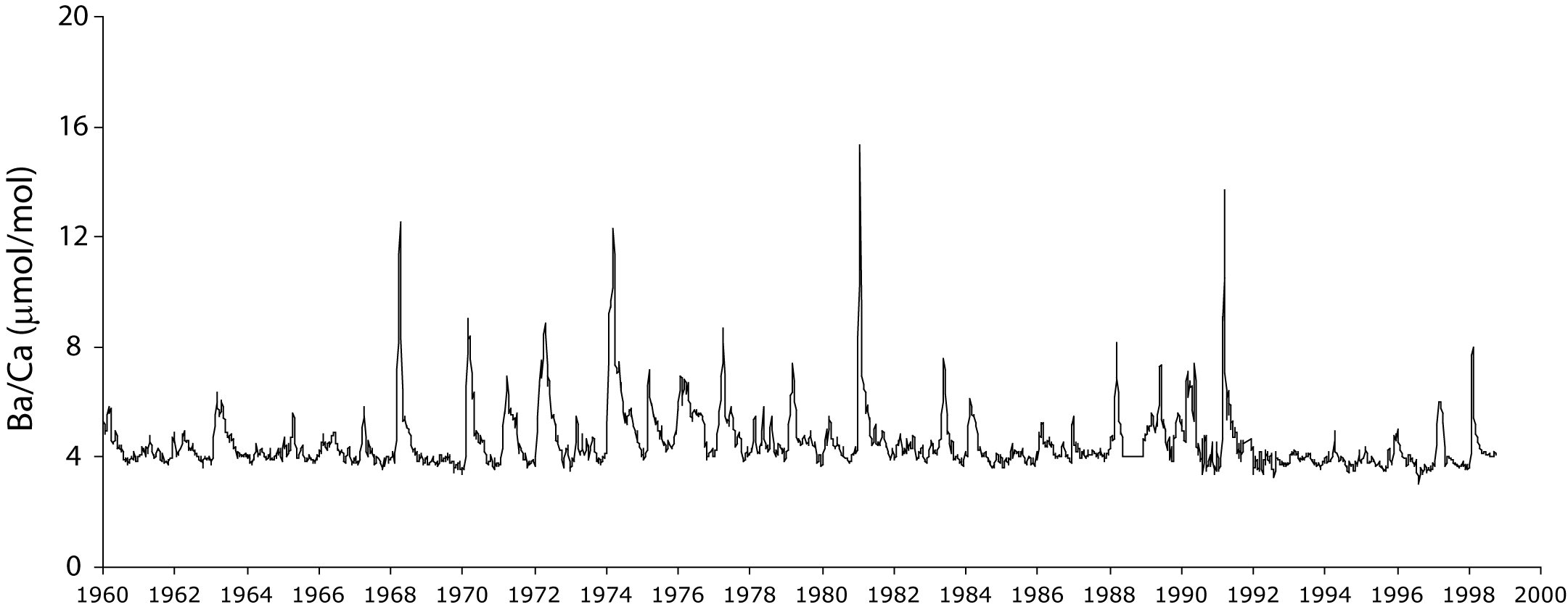

Supplement: Figure S3 — Havannah Ba/Ca timeseries. Portion of previously published [26] compilations of coral records retrieved from other locations at Havannah Island. Some major drought-breaking flooding events are more prominent in these records (e.g. 1968, 1974), likely due to island shadowing of plumes for the record presented in the main text. (TIF) [file pone.0077091.s003.tif]
